# Information seeking about COVID-19 and associated factors among chronic patients in Bahir Dar city public hospitals, Northwest Ethiopia: a cross-sectional study

**DOI:** 10.1186/s12879-022-07315-4

**Published:** 2022-04-01

**Authors:** Sisay Yitayih Kassie, Tesfahun Melese, Simegnew Handebo, Yakub Sebastian, Habtamu Setegn Ngusie

**Affiliations:** 1 Department of Health Informatics, College of Health Sciences, Mettu University, P. O. Box: 318, Mettu, Ethiopia; 2 Department of Health Informatics, Institute of Public Health, College of Medicine and Health Sciences, University of Gondar, Gondar, Ethiopia; 3 Department of Health Education and Behavioural Sciences, School of Public Health, St. Paul’s Hospital Millennium Medical College, Addis Ababa, Ethiopia; 4 College of Engineering, IT and Environment, Charles Darwin University, Casuarina, Australia

**Keywords:** Information seeking, COVID-19 pandemic, Chronic patients, Ethiopia

## Abstract

**Background:**

The health impacts of COVID-19 are not evenly distributed in societies. Chronic patients are highly affected and develop dangerous symptoms of COVID-19. Understanding their information seeking about COVID-19 may help to improve the effectiveness of public health strategies in the future, the adoption of safety measures, and minimize the spread of the pandemic. However, there is little evidence on information seeking specifically on COVID-19 in this study setting. Therefore, this study aimed to assess information seeking about COVID-19 and associated factors among chronic patients.

**Method:**

An institutional-based cross-sectional study supplemented with qualitative data was conducted at Bahir Dar city public hospitals in Northwest Ethiopia from April 8 to June 15, 2021. A total of 423 chronic patients were selected using systematic random sampling techniques with an interval of 5. Bi-variable and multivariable logistic regression analysis was fitted to identify factors associated with information seeking about COVID-19. A p-value < 0.05 was used to declare statistical significance. Qualitative data were analyzed using a thematic approach. Finally, it was triangulated with quantitative findings.

**Result:**

The proportion of information seeking about COVID-19 among chronic patients was 44.0% (95% CI = 39.0, 49.0). Being living in urban [AOR = 4.4, 95% CI (2.01, 9.58)], having high perceived susceptibility to COVID-19 [AOR = 3.4, 95%CI (1.98, 5.70)], having high perceived severity to COVID-19 [AOR = 1.7, 95%CI (1.04, 2.91)], having high self-efficacy to COVID-19 [AOR = 4.3, 95%CI (2.52, 7.34)], and having adequate health literacy [AOR = 1.8, 95%CI (1.10, 3.03)] were significant factors associated with information-seeking about COVID-19.

**Conclusion:**

The overall proportion of information seeking about COVID-19 among chronic patients was low. Thus, health promotion programs should emphasize the chronic patients living in a rural area; enhance perceived risk and severity of COVID-19, enhancing self-efficacy and health literacy interventions to improve information seeking.

**Supplementary Information:**

The online version contains supplementary material available at 10.1186/s12879-022-07315-4.

## Background

Coronavirus disease (COVID-19) is an emerging respiratory disease that causes severe acute respiratory syndrome coronavirus-2 (SARS-CoV-2), which was discovered in Wuhan, China. COVID-19 causes a wide spectrum of morbidity, from moderate respiratory disease to severe consequences such as acute respiratory distress syndrome, septic shock, various metabolic and hemostasis abnormalities, complex severe morbidity, as well as mortality [1]. In December 2019 COVID-19 has declared a global pandemic disease [2, 3]. On January 30, 2020, it was declared as a Public Health Emergency of International Concern by the World Health Organization (WHO) [4]. Then, the virus quickly spread around the world and imposed a critical situation on public health.

The prevalence of COVID-19 progressively increased everyday basis across the world. According to WHO, as of 8 November 2021, 247,743,428 confirmed cases of COVID-19, including 5,047,652 deaths reported in the globe [6]. With political unrest daily new cases of COVID-19 reported in Ethiopia each day are increasingly high: As of 8 November 2021, about 367,210 confirmed cases and 6542 confirmed deaths were reported [7]. Furthermore, the disease significantly affects everyday life, resulting in a socio-economic crisis, and disrupting health service delivery. In low-and middle-income countries due to shortage of health care providers, unable to keep social distancing [9], lack of necessary resources to run the routine health care services delivery. This will make COVID-19 worse and difficult for them to respond to the pandemic. The result could be a catastrophic loss of life [10].

During the pandemic, most studies indicated that patients with underlying diseases not only have a higher risk of developing the disease but also are more likely to die from the virus infection [11]. Patients with chronic cardiac disease, HIV/AIDS, tuberculosis, chronic respiratory disease, and diabetes Mellitus are at the most risk of death in society [12–16]. In Ethiopia, studies indicated that COVID-19 causes 72%, 22.8%, and 1.3% of chronic patients who received poor medication, were psychologically affected, and died respectively [16–18]. Hence, patients need the skill to find, understand, and evaluate the information for self-management and care, engagement to prevent COVID-19 [19–21]. Obtaining information about COVID-19 is necessary at this time to protect one's health from this disease. This approach is crucial for patients to seek information, which is a purposeful search for information to meet their information needs [22].

Information seeking is a conscious effort to acquire information in response to a need or a gap in someone’s knowledge. During the pandemic, information seeking about COVID-19 was acknowledged as a life-saving behavior and adopted as a preventive strategy [23, 24]. In this regard, Ethiopia’s government adopted the strategy and working hard to disseminate information about COVID-19 prevention measures through television, radio, and social media, in addition to declaring a state of emergency and other public health measures [7, 16, 17].

However, studies indicated that seeking health-related information is limited in developing countries including Ethiopia [25–28]. A study conducted in Ethiopia on university students, 69.9% [29], on diabetes patients, 58.4% [30], and a recent study on health care professionals 29.9% [31] did not seek health-related information. Educational status [32–34], age (42), gender [35–37], marital status [38], residence[30], physical activity [39], self-efficacy[40], Perceived susceptibility [26, 29], perceived severity [29], comorbidity status [41], health status (chronic health condition) [39, 42, 43] and health literacy [30, 31, 40] were the major factors influencing health-related information seeking.

In Ethiopia, the evidence on health-related information seeking and information use culture were low [44]. However, to the best of our knowledge, there is limited evidence on information seeking about COVID-19 among chronic patients in low-income countries. Investigating and identifying patients' information seeking about COVID-19 enables to support the development of useful strategies for effective information transfer of patients, thereby improving patient self-management and preventing the pandemic. Therefore, this study aimed to assess information seeking about COVID-19 and associated factors among chronic patients.

## Method

### Study design and setting

An institutional-based cross-sectional study supplemented with the qualitative study was conducted from April 8 to Jun 15 in 2021 among chronic patients with chronic disease at Bahir Dar city public hospitals. The Bahir Dar city, the capital city of Amhara National Regional State is located about 570 km northwest of Addis Ababa. The city has 9 sub-cities and 12 rural kebele with a total population of 321,000. The city has one specialized teaching hospital, one comprehensive specialized referral hospital, and one primary hospital. Currently, there are 19,677 chronic patients attending care in these public hospitals.

### Study participants

The study participants were all patients with chronic disease who are attending health care at Bahir Dar city Public Hospitals. All selected chronic patients with HIV/AIDS, Tuberculosis, Diabetes, chronic respiratory disease, chronic cardiovascular disease aged above 18 years, and those who had a follow-up in Bahir Dar city public hospitals were included in the study. Patients whose age was below 18 years and who could not able to give responses owing to serious illness at the time of data collection were excluded from the study.

### Sample size determination

The sample size was determined using a single population proportion formula considering the following assumptions P (50%, proportion of chronic patients who seek COVID-19 related information (since there is no previous study), d (the permissible margin of error 5%), and Zα/2 (the value of the standard normal curve score corresponding the given confidence interval 1.96) corresponding to 95% confidence level.

$$n={ ({z}_{a/2})}^{2}\frac{p (1-p)}{{d}^{2}}$$

Thus, after adjusting for the non-response rate (10%), the final sample (n) was 423. For the qualitative approach, the sample size was determined based on information saturation. Accordingly 5 chronic patients and 3 health professionals were interviewed.

### Sampling method and procedure

A stratified sampling followed by a systematic random sampling technique was used to recruit study participants. The selected patients with chronic diseases were interviewed using a structured Amharic language-version questionnaire. During the data collection period, the interval size (K) was calculated first by the following formula: K = N/n.

Where K = the interval size, N = total average number of selected chronic patients who visit per month the hospitals (2,191) in Bahir Dar city public hospitals, and n = number of sampled chronic patients (423). Thus, Kth = 5 (select every 5th unit) using patients’ records, which listed the follow-up appointments. Then an integer (5) was randomly selected through the lottery method between 1 and k (5), and every kth (5) unit was taken. Finally, 423 chronic patients were approached. The detail schematic presentation is found in Additional file 1.

For the qualitative study, a participant was selected using a purposive sampling technique (criteria-based). Criteria for selecting chronic patients were educational level, residence, age, and key informants.

### Measurement

#### Perceived susceptibility to COVID-19

Refers a person’s subjective perception of the risk of acquiring COVID-19 and measured by four-item question with five-point Likert scale ranges from strongly disagree (1) to strongly agree (5). The higher summed score indicates higher perceived susceptibility towards COVID-19.

#### Perceived severity

Refers to a person's impression of the seriousness of contracting COVID-19, which is tested using four items on a five-point Likert scale ranging from strongly disagree (1) to strongly agree (5). The higher the total score, the more severe COVID-related symptoms are assessed to be.19.

#### Self-efficacy

Refers to the confidence level of a person has about their capacity to undertake behavior (s) that may lead to desired outcomes towards COVID-19 preventive practices and measured by four items having a five-point Likert scale it ranges from strongly disagree (1) to strongly agree (5) adapted from respective literature.

#### Health literacy towards COVID-19

The European Health Literacy Survey Questionnaire was used to assess health literacy related to the Coronavirus (HLS-EU-Q). The HLS-EU-Q is a self-report tool that assesses a broad model of health literacy [45]. It measures how difficult or easy it is for participants to access, comprehend, appraise, and use health information. Originally, the HLS-EU-Q included 47 components (HLS-EU-Q47), but abbreviated forms have been developed throughout time. The 16-item version is often known as the (HLS-EU-Q16) [46]. During the coronavirus pandemic, 22 items were produced (HLS-COVID-Q22), which are structured into four subscales: accessing (six items), understanding (six items), assessing (five things), and applying (five items) health-related information. Index = mean-1* (50/3) is the formula for calculating an index. If the index score is less than 2.5, it means you have “inadequate health literacy,” if the index score is between 2.5–3 means you have “problematic health literacy,” and 3 means you have “sufficient health literacy”. We used the median as a cut-off in this study because of the data distribution, and participants who scored above the median had adequate health literacy.

#### Smoker

Is a respondent who smokes currently either every day or sometimes.

#### COVID-19 information seeking about COVID-19

And measured by reviewing the existing literature on information seeking about COVID-19[29–31]. The respondents were asked. Have you ever sought information about COVID-19 purposely/intentionally during the past 6 months from different sources such as health professionals, the Internet, mass media, family, textbooks and/or brochures, and “Yes” = respondents sought information and “no” otherwise, and codded 1 and 0 respectively.

### Data collection tool

A structured, translated, interviewer-administered Amharic-version questionnaire which was developed from relevant literature comprised of demographic, behavioral, psychological, and health-related factors was used [29–31, 47]. Coronavirus-related health literacy was assessed based on the European Health Literacy Survey Questionnaire (HLS-COVID-Q22) [48, 49]. COVID-19 information-seeking questions are mainly adapted from the Health Information National Trend survey 2014. Initially, it was developed in the English language and translated to Amharic by language experts. The Amharic questionnaire was re-translated back to English to check the consistency of translation. A pretest was conducted on 5% of the sample at the University of Gondar specialized Referral Hospital. The tool was revised based on the findings of the pre-test. Two BSc nurse and two MPH students were assigned to collect the data and for supervision, respectively. A two days training was given for the data collectors and supervisors on the objective of the study, data collection method, the content of the questionnaire, and ethical issues.

For the qualitative study, participants were selected purposely from those who were willing to participate in a semi-structured in-depth interview guide was developed by the investigator to prompt the discussion and elicit details through probing. Finally, in-depth interviews among 5 patients with chronic disease and 3 key informants were conducted by the principal investigator, using the Amharic language for discussion.

### Data processing and analysis

The completed questionnaire was checked for consistency and completeness and entered into EpiData version 4.6 and exported to SPSS version 25 for analysis. Descriptive statistics and binary logistic regression analysis were used to describe the socio-demographic characteristics of patients with chronic diseases and to identify factors associated with information-seeking about COVID-19, respectively. Bi-variable and multivariable analyses were conducted to identify the associations between each independent variable and the dependent variables. P values < 0.2 and < 0.05 were taken as cut-off values in significance tests for the bi-variable and multivariable analyses, respectively.

A qualitative study was conducted by the investigator using open-ended questions for identifying barriers and challenges on information-seeking about COVID-19 that could not be answered using the quantitative method. An in-depth interview guide with a tape recorder was used for gathering qualitative data. Qualitative data were collected using a tape recorder and the recorded data were transcribed in the English language. The transcribed data were coded and categorized into themes. Finally, thematic content analysis was conducted to identify emerging themes and support the quantitative findings.

## Result

### socio-demographic characteristics

A total of 423 chronic disease patients were interviewed with a response rate of 100%. Among this 152 (35.9%) were HIV/AIDS patients followed by Diabetics 148 (35.0%). The mean age of the study participants was 45.2 years (SD ± 13.4 years). More than half of them, 227 (53.7%) were females. The majority were urban dwellers 332 (78.5%) and Orthodox Christians 372 (87.9%). About two-thirds (67.1%) of the respondents were married. Regarding educational status, 143 (33.8%) had higher educational status and 66 (21.5%) had primary education (Table 1).

**Table 1:** Socio-demographic characteristics of chronic patients in Bahir Dar city public hospitals, Ethiopia, 2021 (n = 423)

Variables	Frequency	Percentage
Chronic patients		
HIV/AIDS	152	35.9
Diabetes	148	35.0
Chronic respiratory disease	31	7.3
Chronic cardiac disease	89	21.1
TB	3	0.7
Gender		
Female	227	53.7
Male	196	46.3
Age (mean &SD)	45.2 ± 13.4	
Marital status		
Single	71	16.8
Married	284	67.1
Divorced	32	7.6
Widowed	36	8.5
Religion		
Orthodox	372	87.9
Others	51	12.1
Residence		
Urban	332	78.5
Rural	91	21.5
Occupation		
Farmer	57	13.5
Government employee	121	28.6
Housewife	70	16.5
Merchant	62	14.7
Daily labor	36	8.5
Student	19	4.5
Unemployed	20	4.7
Others	38	9.0
Education status		
Non-formal education	91	16.3
Primary education	66	21.5
Secondary education	54	12.8
Higher education	143	33.8

### Behavioral characteristics

Out of the study participants, 68 (16.1%) were doing physical exercise & 15 (2.0%) were physically active and Only 10 (2.4%) were smoking cigarettes.

### Psychological characteristics

From a total study participants’, 192 (45.4%) perceived that they were susceptible to COVID-19 and 186 (44.0%) also perceived that the disease was severe. On the other hand, nearly half, 205 (48.5%) were confident to take behavior to overcome COVID-19 (Table 2).

**Table 2:** Psychological characteristics of chronic patients in Bahir Dar city public hospitals Ethiopia, 2021 (n = 423)

Variables	Frequency	Percentage
Perceived susceptibility		
Not susceptible	231	54.6
Susceptible	192	45.4
Perceived severity		
Not severe	237	56.0
Severe	186	44.0
Self-efficacy		
Not confident	218	51.5
Confident	205	48.5

### health-related characteristics

Among the study participants, 156 (36.9%) were living with co-morbidity. The median duration of illness was 6 years with an inter-quartile range (± 3.6 years). Half 214 (50.6%) of the study participants had adequate health literacy (Table 3).

**Table 3:** Health-related characteristics of chronic patients in Bahir Dar city public hospitals Ethiopia 2021 (n = 423)

Variables	Frequency	Percentage
Duration of illness (media & IQR)	6 ± 3.6	
Co-morbidity		
Yes	156	36.9
No	267	63.1
Health literacy		
Limited	209	49.4
Adequate	214	50.6

### Information-seeking about COVID-19

**Fig. 1:**
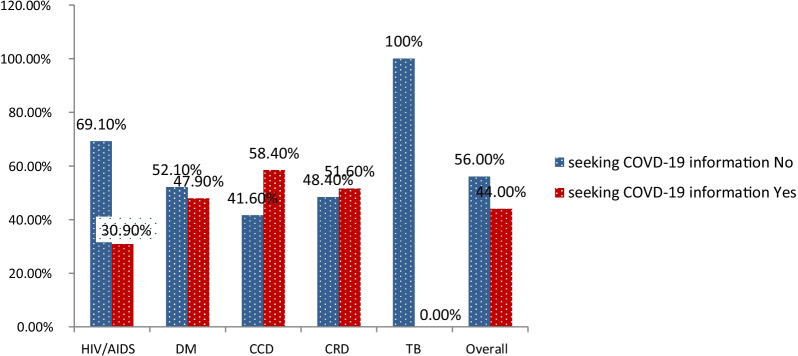
Information seeking about COVID-19 among each type of chronic diseases patients in Bahir Dar City public hospitals, Ethiopia 2021

Of the total, 44.0% (95% CI = 39.0, 49.0) of study participants have sought information about COVID-19 purposively/intentionally in the past 6 months. However, 237 (56.0% (95% CI)) have not sought information about COVID-19 purposively or intentionally in the past 6 months (Fig. 1). The result of the qualitative study indicates that misinformation about the disease and repeated messages, negative perception of neighbors, families, and religious leaders, and low educational status and awareness were the major hindering barriers to COVID-19 information seeking.

Misinformation about the COVID-19 and repeated similar messages from the sources were the main factors that hinder information seeking about COVID-19 among chronic patients. A participant explained this as below:

“I did not seek information about COVID-19 from digital technologies. This is due to misinformation spread about the disease from digital technologies.” (A 27 years old asthmatic patient 1).

Other participants support the above idea:

“I am not seeking information about COVID-19 currently. This is because all the information was repeated; nothing is new about the pandemic.” (A word of 37 years old with higher educational status 4).

The negative perception of neighbors, families and religious leaders towards the disease were the main barriers and challenges to give more concern and hindering factors to seek information about COVID-19. A participant explained this as follows.

“It's a challenge for me not to be overly concerned about the disease and seek information about it from various sources because my neighbors and family have a negative perception about the pandemic” (A word of 27 years old with higher educational status 2).

A key informant support the above idea.

“In any case, the disease related-information is the same. It is all about halting the disease spread. I give the same information as my partners; as a result, they develop negative perception during information campaign about the disease.” (A word of key informant 3).

Low educational status is another factor for low COVID-19 related information seeking among chronic patients from information sources. A participant explains this as follows.

“I can’t seek additional information than the usual from the internet about the pandemic. Due to my education status and this forces me to give low attention for the disease.” (A word of rural residence 40 years old HIV/AIDS patient 3).

A key informant supports the above idea:

“Most patients did not ask a question occasionally about the disease from health care professionals to care themselves. This is due to low educational status.” *(A word of key informant 2).*

### Distribution of measurement scales with each type of chronic disease patients

From a total of 152 HIV/AIDS study participants, 131 (86.2%) were from urban residence, 65 (42.8%) have adequate health literacy, 70 (46.0%) were susceptible to COVID-19, 49 (32.2) were perceived that COVID-19 is severe, and 55 (36.2) of them were confident to take behavior to prevent the disease.

From the study 148 Diabetic patients were participated among from this 67 (75.7%) were from urban residence, 77 (52.0%) of them were perceived that COVID-19 is severe, 73 (49.3%) were perceived that they were susceptible to developing COVID-19, and 73 (49.3%) have adequate health literacy. Of 423 study participants, 89 of them were chronic cardiac patients. From this, 67 (75.3%) were from urban residence, 57 (64.0%) were confident to take behavior, 56 (62.9%) have adequate health literacy, 40 (44.9%), and 50 (56.2%) were perceived susceptible and severe respectively.

Of 31 chronic respiratory diseases study participants, 21 (67.7%) were from urban residences. Regarding health-related factors, 19 (61.3%) of them have adequate health literacy and 12 (38.7%) were living with additional comorbidity. And also 9 (29.0%) and 10 (32.3%) chronic respiratory patients perceived that they were susceptible to developing COVID-19 and perceived that COVID-19 were severe. However, 20 (64.5%) of them were confident to take behavior to prevent the disease (Table 4).

**Table 4:** The distribution of measurement scales among each type of chronic disease patients who attend their follow-up in Bahir Dar City Public Hospitals, Ethiopia 2021

Measurement scales	HIV/AIDS (%)	DM (%)	CCD (%)	CRD (%)	TB (%)
Sex					
Female	95 (62.5)	65 (43.9)	49 (55.1)	16 (51.6)	2 (66.7)
Male	57 (37.5)	83 (56.1)	40 (44.9)	15 (48.4)	1 (33.3)
Residence					
Urban	131 (86.2)	112 (75.7)	67 (75.3)	21 (67.7)	1 (33.3)
Rural	21 (13.8)	36 (24.3)	22 (24.7)	10 (32.3)	2 (66.7)
Educational status					
Non-formal education	49 (32.2)	61 (41.2)	37 (41.6)	10 (32.2)	3 (100)
Primary education (1–8)	37 (24.3)	20 (13.5)	6 (6.7)	3 (9.7)	0.0
Secondary education (9–12)	29 (19.2)	8 (5.4)	15 (16.9)	2 (6.5)	0.0
Higher education	37 (24.3)	59 (39.9)	31 (34.8)	16 (51.6)	0.0
Smoking cigarettes					
Yes	3 (2.0)	4 (2.7)	1 (1.1)	2 (6.5)	0 (0.0)
No	149 (98.0)	144 (97.3)	88 (98.9)	29 (93.5)	3 (100)
Health literacy					
Limited	87 (57.2)	75 (50.7)	33 (37.1)	12 (38.7)	2 (66.7)
Adequate	65 (42.8)	73 (49.3)	56 (62.9)	19 (61.3)	1 (33.3)
Perceived susceptible					
Not susceptible	82 (54.0)	75 (50.7)	49 (55.1)	22 (71.0)	3 (100.0)
Susceptible	70 (46.0)	73 (49.3)	40 (44.9)	9 (29.0)	0 (0.0)
Perceived severity					
Not severe	103 (67.8)	71 (48.0)	39 (43.8)	21 (67.7)	3 (100.0)
Severe	49 (32.2)	77 (52.0)	50 (56.2)	10 (32.3)	0 (0.0)
Self-efficacy					
Not confident	97 (63.8)	76 (51.3)	32 (36.0)	11 (35.5)	2 (66.7)
Confident	55 (36.2)	72 (48.7)	57 (64.0)	20 (64.5)	1 (33.3)
Co-morbidity					
Yes	30 (19.7)	76 (51.3)	36 (40.4)	12 (38.7)	2 (66.7)
No	122 (80.3)	72 (48.7)	53 (59.6)	19 (61.3)	1 (33.3)
Marital status					
Single	33 (21.7)	10 (6.7)	24 (27.0)	4 (12.9)	0 (0.0)
Married	89 (58.6)	112 (75.7)	58 (65.2)	23 (74.2)	2 (66.7)
Divorced	19 (12.5)	9 (6.1)	2 (2.2)	1 (3.2)	1 (33.3)
Widowed	11 (7.2)	17 (11.5)	5 (5.6)	3 (9.7)	0 (0.0)

### Factors associated with information-seeking about COVID-19

In the bivariate analysis, socio-demographic, behavioral, psychological, and health-related factors were associated with information-seeking about COVID-19 at a *P-value* < 0.2 and were entered in the multivariable analysis. The multivariable logistic regression further revealed that living in urban, perceived susceptibility, perceived severity, self-efficacy, and health literacy were statistically significant factors associated with information-seeking about COVID-19 at a p-value < 0.05.

Chronic patients living in urban areas were 4.4 times [AOR = 4.4, (95% CI: 2.01, 9.58)] more likely to seek information about CVID-19 than those living in rural areas. Chronic patients who were perceived susceptible to developing COVID-19 were about 3.4 times more likely to seek information about COVID-19 compared to their counterparts [AOR = 3.4, (95% CI: 1.98, 5.70)]. Similarly, chronic patients who perceived COVID-19 as severe were 1.7 times more likely to seek information about COVID-19 compared to those who perceived COVID-19 as not severe [AOR = 1.7, (95%CI: 1.04, 2.91)].

Self-efficacy was also found to be a factor that significantly associated with information-seeking about COVID-19. Thus, chronic patients who were confident to execute or undertake the behavior to reduce the risk of developing COVID-19 were 4.3 times [AOR = 4.3, (95%CI: 2.52, 7.34)] more likely to seek information about COVID-19 compared to those chronic patients who were not confident to take behavior or actions to reduce the risk of developing COVID-19.

Furthermore, another factor that is statically significantly associated with information-seeking about COVID-19 is health literacy. Chronic patients who have adequate health literacy were 1.8 times [AOR = 1.8, (95%CI: 1.10, 3.03)] more likely to seek information about COVID-19 than those who have limited health literacy. The output of the logistic regression analysis is presented in Table 5.

**Table 5:** Bivariate and multivariate analysis of factors associated with information-seeking about COVID-19 among chronic patients in Bahir Bar city public hospitals, Ethiopia 2021 (n = 423)

Variables	Category	Information-seeking about COVID-19		COR (95%CI)	AOR (95%CI)
		No (n = 237)	Yes (n = 186)		
Age (Mean + SD)	(45.2 ± 13.4)			0.87 (0.67,0.99)	0.56 (0.48,1.03)
Sex	Female	129 (54.4)	98 (52.7)	1	1
	Male	108 (45.6)	88 (47.3)	1.07 (1.03,1.98)	0.7 (0.42,1.25)
Residence	Urban	156 (65.8)	176 (94.6)	9 (4.58,18.25)	4.4 (2.01,9.58)***
	Rural	81 (34.2)	10 (5.4)	1	1
Marital status	Single	30 (12.7)	41 (22.0)	1	1
	Married	160 (67.5)	124 (66.7)	0.6 (0.34,0.96)	0.8 (0.36,1.63)
	Divorced	21 (8.9)	11 (5.9)	0.4 (0.16,0.91)	0.7 (0.24,2.22)
	Widowed	26 (11.0)	10 (5.4)	0.3 (0.12,0.67)	0.7 (0.19,2.23)
Perceived susceptibility	Not susceptible	161 (67.9)	70 (37.6)	1	1
	Susceptible	76 (32.1)	116 (62.4)	3.5 (2.35,5.25)	3.4 (1.98,5.70)***
Perceived severity	Not severe	160 (67.5)	77 (41.4)	1	1
	Severe	77 (32.5)	109 (58.6)	2.9 (1.97,4.38)	1.7 (1.04, 2.91)*
Self-efficacy	Not confident	163 (68.8)	55 (29.6)	1	1
	Confident	74 (31.2)	131 (70.4)	5.2 (3.45,7.97)	4.3 (2.52,7.34)***
Health literacy	Limited	152 (64.1)	57 (30.6)	1	1
	Adequate	85 (35.9)	129 (69.4)	4 (2.69,6.10)	1.8 (1.10,3.03)*

*p-value > 0.01, p-value < 0.01*** and 1 reference category

## Discussion

This research assessed information seeking about COVID-19 and associated factors among chronic patients in Northwest Ethiopia. In this study, 44.0% (95% CI = 39.0, 49.0) of participants responds they sought information about COVID-19 in the past six months before the survey. This revealed that the majority of chronic patients have not sought information about COVID-19. Results from the qualitative study support this low proportion of information seeking about COVID-19 was mainly due to misinformation and repeated information from the source, low educational status, negative perception of neighbors, families, and religious leaders towards COVID-19. This result is lower than that reported in a study conducted in Iran (75.2%)[21]. The possible explanation for this discrepancy in the result might be due to the internet penetration rate. According to the 2021 World Internet Statistics report, the internet penetration rate in Ethiopia is 17.9%, whereas the internet penetration rate in Iran is 58.4%, respectively. A high internet penetration rate might help individuals can easily find, access, and search health-related information from information sources.

Besides, this finding is also lower than a study conducted in Ethiopia, at the University of Gondar specialized teaching hospital among health care providers (71.1%) [31]. This disparity might be due to the difference in study participants’ level of health literacy and the study period. The study done in Ethiopia was conducted during the lockdown when there was high demand for information about COVID-19. Another possible reason might be due to differences in study participants. The research study in Ethiopia was conducted among health care professionals who had higher educational levels and health literacy as compared to chronic patients.

Information seeking about COVID-19 among chronic patients in this study is nearly similar to that reported by a study conducted in Ethiopia (41.6%) [30]. The possible explanation for this might be due to similar socio-demographic characteristics of the study participants and equal sample size. In the research study conducted in Debremarkos hospital among diabetic patients, the socio-demographic characteristics of the study participants are nearly similar compared to chronic patients who attained their follow-up in Bahir Dar city public hospitals. In contrast, our finding was higher than a study conducted in Egypt 22.2% [43]. This might be due to the difference in study participants and the risk of perception to develop the COVID-19. In this study, the participants were chronic patients who are more vulnerable and have higher death as compared to the general population.

Information seeking about COVID-19 was interlinked with socio-demographic, psychological, and health-related factors. In this study finding, the residence of chronic patients is significantly associated with information-seeking about COVID-19. Chronic patients who lived in urban areas were more likely to seek information about COVID-19 compared to rural areas. This is supported by previous research study findings [30]. This might be due to increased access to mass media daily, social network, and a mobile phone that helps them to find information about the disease easily. Another possible explanation might be fear of developing the disease since the prevalence of COVID-19 is higher in an urban area.

Chronic patients who were perceived susceptible to developing the disease were more likely to seek information about COVID-19. This was supported by previous research findings [26, 29]. This could be due to the perceived susceptibility to develop the disease initiate to look for information measures to reduce their risk of developing and preventing the problem. Results of the previous study support this finding that individuals who are concerned about getting health problems are more likely to look for health-related information than those who are not concerned about getting health problems [26]. This research finding strengthens the notation that a high perception of disease susceptibility is inter-related with high information seeking.

Chronic patients who perceived COVID-19 as severe were more likely to seek information about COVID-19. This finding was supported by previous research finding in Ethiopia [29]. This is because if the disease severity increased individuals need to know the possible risk factors, prevention methods, treatments, and disease complications of COVID-19 with their previous disease. This finding is supported by a qualitative finding.

A key informant 2 said “Not all, but most of them tend to look health information and asks health professionals on the nature of the disease when they face severe.”

Furthermore, self-efficacy was found significantly associated with information-seeking about COVID-19. Participants who were confident to take behavior to reduce the risk of developing the disease were more likely to seek information about COVID-19. This finding is supported by a research finding [40].

Health literacy is a factor found to influence information seeking about COVID-19. Chronic patients who have adequate health literacy were more likely to seek information about COVID-19 than those who have limited health literacy. This study finding is consistent with the previous studies which state that individuals who have adequate health literacy were more likely to engage about their health to understand treatments about their health problems and critically appraise information they seek from different sources [26, 29, 30]. This finding was supported by previous research studies.

This finding revealed that adequate health literacy level has a direct relationship with health-related information seeking. This finding is supported by the qualitative finding.

A key informant 3 said “Patients who had good health literacy ask a question about their health condition. But in number they are small.”

## Conclusion

Overall, the proportion of information seeking about COVID-19 was low among chronic patients in the past 6 months. In this study, urban residence, higher health literacy level, perceived susceptibility, perceived severity, and higher self-efficacy were factors significantly associated with information-seeking about COVID-19. Signifying adequate health literacy level and self-efficacy directed at improving information seeking about COVID-19 and useful for combating the pandemic and continuing safe practices among chronic patients.

## Strengths and limitations of the study

This study was assessing information seeking about COVID-19 among chronic patients and supported by qualitative findings. However, this study uses an interviewer self-administer questionnaire to collect the data and will lead to an interviewer bias. Moreover, this study shares the limitation of cross-sectional studies, therefore may not provide a strong cause-effect relationship.

## Supplementary Information

**Additional file 1.** Schematic presentation of the sampling procedure.

## Data Availability

The datasets generated and/or analyzed during the current study are not publicly available due to some of the study participant’s data being sensitive but are available from the corresponding author on reasonable request.

## Abbreviations

AOR: Adjusted odds ratio
CI: Confidence intervals
COR: Crude odds ratio
HIV/AIDS: Human immunodeficiency virus
SPSS: Statistical package for social science
WHO: World Health Organization
