# Supplementary material for: Information seeking about COVID-19 and associated factors among chronic patients in Bahir Dar city public hospitals, Northwest Ethiopia: a cross-sectional study
Source: BMC Infect Dis. 2022 Apr 1;22:325. doi: 10.1186/s12879-022-07315-4 (PMC8972753; doi:10.1186/s12879-022-07315-4)
Supplement: Supplementary file 1 — Additional file 1. Schematic presentation of the sampling procedure. [file 12879_2022_7315_MOESM1_ESM.pdf]

## Schematic presentation of the sampling procedure

To select the study subjects, a stratified sampling technique followed by a systematic random sampling technique was employed. (chronic cardiovascular disease, TB, HIV/AIDS, chronic respiratory disease and Diabetes Mellitus) from 19,677 chronic patients in the hospitals. . Based on proportional allocation, 423 chronic patients were selected.

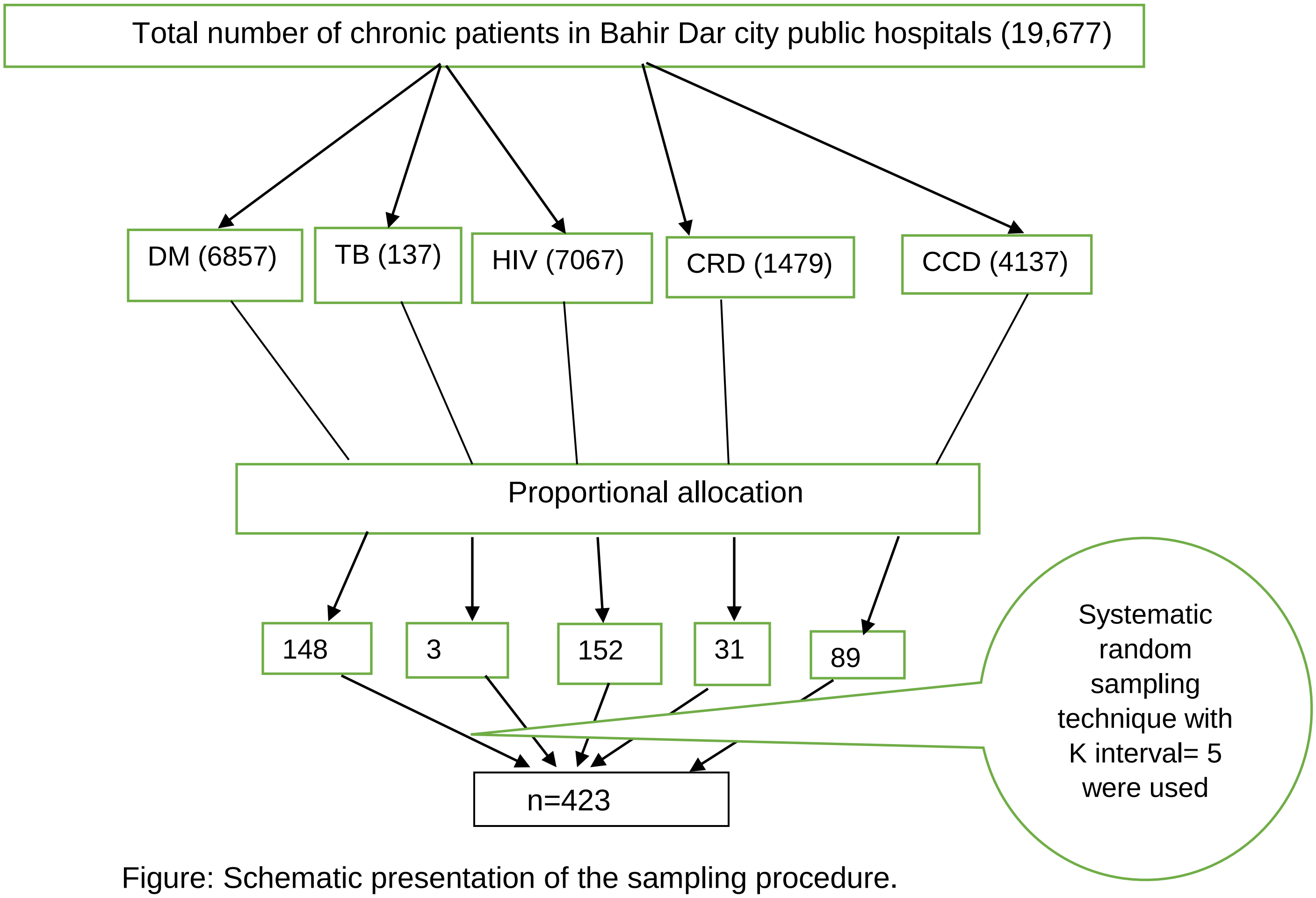


Figure: Schematic presentation of the sampling procedure.

During the data collection, the skipping interval was determined by dividing the estimated number of chronic patients who take service per month by the respective sample size. Hence, each participant was selected by systematic random sampling with a skip interval of 5 using the lottery method. 5 was used as a starting number. Subsequently, every 5$^{th}$ patient was included in the study.

$$K^{th} = \frac{N}{n} = \frac{2,191}{423} = 5.17 = 5^{th}$$

Where

N= total average number chronic patients who visit per month (2,191)

n= sampled chronic patients (423)

$K^{th}$=5 (select every $5^{th}$ unit
